# Advances in the quantification of mitochondrial function in primary human immune cells through extracellular flux analysis

**DOI:** 10.1371/journal.pone.0170975

**Published:** 2017-02-08

**Authors:** Dequina Nicholas, Elizabeth A. Proctor, Forum M. Raval, Blanche C. Ip, Chloe Habib, Eleni Ritou, Tom N. Grammatopoulos, Devin Steenkamp, Hans Dooms, Caroline M. Apovian, Douglas A. Lauffenburger, Barbara S. Nikolajczyk

**Affiliations:** 1 Department of Microbiology, Boston University School of Medicine, Boston, MA, United States of America; 2 Department of Biological Engineering, Massachusetts Institute of Technology, Cambridge, MA, United States of America; 3 Department of Medicine, Boston University School of Medicine, Boston, MA, United States of America; 4 BioEnergetics LLC, Boston, MA, United States of America; 5 Arthritis Center, Boston University School of Medicine, Boston, MA, United States of America; 6 Department of Pathology, Boston University School of Medicine, Boston, MA, United States of America; 7 Department of Molecular and Cell Biology, Boston University School of Medicine, Boston, MA, United States of America; Hospital for Sick Children, CANADA

## Abstract

Numerous studies show that mitochondrial energy generation determines the effectiveness of immune responses. Furthermore, changes in mitochondrial function may regulate lymphocyte function in inflammatory diseases like type 2 diabetes. Analysis of lymphocyte mitochondrial function has been facilitated by introduction of 96-well format extracellular flux (XF96) analyzers, but the technology remains imperfect for analysis of human lymphocytes. Limitations in XF technology include the lack of practical protocols for analysis of archived human cells, and inadequate data analysis tools that require manual quality checks. Current analysis tools for XF outcomes are also unable to automatically assess data quality and delete untenable data from the relatively high number of biological replicates needed to power complex human cell studies. The objectives of work presented herein are to test the impact of common cellular manipulations on XF outcomes, and to develop and validate a new automated tool that objectively analyzes a virtually unlimited number of samples to quantitate mitochondrial function in immune cells. We present significant improvements on previous XF analyses of primary human cells that will be absolutely essential to test the prediction that changes in immune cell mitochondrial function and fuel sources support immune dysfunction in chronic inflammatory diseases like type 2 diabetes.

## Introduction

Immune cells are main sources of the inflammation that supports obesity-associated insulin resistance and type 2 diabetes (T2D) [1, 2]. Lymphocytes such as T cells and B cells contribute to obesity-associated inflammation in unhealthy adipose tissue [3–6], but the paucity of lymphocytes, and especially B cells, in human adipose tissue remains a challenge that limits functional and mechanistic studies on these cells. Several lines of evidence indicate that blood lymphocytes are a reasonable surrogate to guide studies aimed at understanding the roles T cells and B cells play in obesity-associated complications like insulin resistance and T2D [7–13]. These studies include our recently published T cell cytokine signature, which distinguishes samples from T2D and body mass index-matched non-T2D subjects, and was developed from analysis of peripheral blood mononuclear cells [14].

Many recent insights in the field of immunometabolism have focused on roles immune cells play in obesity-associated inflammation, but parallel development of the more traditional branch of immunometabolism aimed at understanding the generation of ATP for immune responses has also accelerated over the past decade [15]. Fuel sources and fuel utilization are now recognized as key regulators of immune responses that include CD4^+^ T cell and macrophage subset skewing, memory T cell formation/maintenance and B cell function [16–22]. These studies include demonstrations that inflammatory T effector subsets such as Th1, Th2, and Th17 cells, and inflammatory M1 macrophages express high amounts of the glucose transporter GLUT1 upon activation to facilitate uptake of the glucose that disproportionately provides ATP through anaerobic glycolysis. In contrast, anti-inflammatory, regulatory CD4^+^ T cells (Tregs) and tissue-remodeling M2 macrophages rely on fatty acid oxidation to drive the oxidative phosphorylation that these cells require for function [21, 23–29]. The field has not tested the possibility that shifts in the nutrient milieu that immerses immune cells in obesity/T2D, alone or in combination with cell-intrinsic changes in fuel utilization, mechanistically explain the compromised immune function in such subjects leading to impaired wound healing and pathogen clearance.

Many conceptual advances in the understanding of fuel utilization by immune cells from non-obese/T2D individuals have been supported by extracellular flux (XF) analysis, which measures oxygen consumption rate (OCR) and/or lactate production (as measured by extracellular acidification rate/ECAR) as indicators of aerobic glycolysis/oxidative phosphorylation or anaerobic glycolysis, respectively. Technical details and interpretive value of this approach have been well described [30, 31]. The advantage of XF analysis is that single wells seeded with relatively few cells can inform investigators on a variety of measures of mitochondrial function including basal respiration, ATP production, proton leak, maximal respiration, spare respiratory capacity and non-mitochondrial respiration with relatively high throughput. Although many publications have highlighted XF analysis of primary human T cells [32–36], the variety of conditions used by investigators to measure mitochondrial function makes comparison amongst studies challenging. Furthermore, limitations in the analytical software included limits on the number of samples that can be combined to assess biological variability, and manual data manipulations and lack of objective quality control steps that could inadvertently introduce error. These limitations significantly compromise utility of XF, especially given the inherent variability of human samples. Detailed standardization of XF protocols and more objective, flexible analytical approaches are absolutely essential to test the prediction that changes in fuel sources in obesity/T2D, coupled with disease-associated changes in immune cell function, combine to mechanistically explain the chronic inflammation, inefficient pathogen clearance and defects in wound healing that plague people with T2D.

## Materials and methods

### Cells

Human samples were obtained following written informed consent under Boston University Institutional Review Board-approved protocols (H27007; H32371) in accordance with the Declaration of Helsinki. Study design was cross-sectional and automation of the analysis minimized the possibility of operator-associated bias. T2D and, as a comparative cohort, type 1 diabetes (T1D) subjects were recruited from the Endocrinology clinic and the Center for Endocrinology, Diabetes and Nutrition at the Boston University Medical Center (BUSM). Additional T2D and systemically healthy obese or lean subjects were recruited from the Clinical Research Center and the BUSM community. Characteristics of subjects are shown in Table 1; none of these parameters (age, BMI, etc.) were statistically different between T1D and T2D cohorts. All subjects were non-smokers. Fifty milliliters peripheral blood was collected into ACD tubes by venous puncture. PBMCs or CD4^+^ T cells were purified by histopaque 1077 followed by negative selection with CD4^+^ cell-excluding magnetic beads (Miltenyi) for experiments on purified T cells as we published [10]. B cells were purified by negative selection based on CD19 expression [37]. Only cell preparations that were >90% pure as re-analyzed by flow cytometry for the appropriate markers were used in analyses on purified cells.

**Table 1:** Description of subjects.

	T1D	T2D
Total N	6	11
Age, years [median (range)]	39.5 (19–59)	50 (41–60)
A1c, % [median (range)]	7.7 (6–12.8)	7.9 (6.1–11.9)
A1c, (mmol/mol) [median (range)]	61 (42–116)	63 (43–107)
BMI, kg m^-2^ [median (range)]	32 (24–37)	35 (29–40)
Random Glucose, mg dL^-1^ [median (range)]	168 (123–588)	151.5 (100.0–266.0)
Systolic Blood Pressure, mmHg [median (range)]	111.5 (101–158)	125 (106–137)
Diastolic Blood Pressure, mmHg [median (range)]	74 (64–93)	78 (65–90)
HDL, mg dL^-1^ [median (range)]	55 (46–71)	44 (40–131)
LDL, mg dL^-1^ [median (range)]	96 (58–190)	81 (48–127)
Females	5	7
Males	1	4

### T cell activation

CD4^+^ T cells were activated using human T activator αCD3/CD28 Dynabeads unless otherwise noted. Dynabeads were washed in PBS, pH 7.4 + 0.05% BSA + 1.0 mM EDTA then retained on a magnet. After removing wash buffer, Dynabeads were resuspended to the original volume in cell culture media. Cells were activated with ~2μL Dynabeads per 100k cells for 40 hrs. Following activation, Dynabeads and any lingering bead-associated cells were removed with a magnet and bead-free cells were analyzed in the XF96 analyzer as described below. Supernatants were saved for cytokine analysis. Cells were frozen either pre- or post-stimulation at -80°C under controlled cooling conditions in a Mr. Frosty apparatus (Nalgene). For multi-week storage, cells were moved to -170°C following 1–7 days at -80°C.

### Metabolic assays

**Fig 1:**
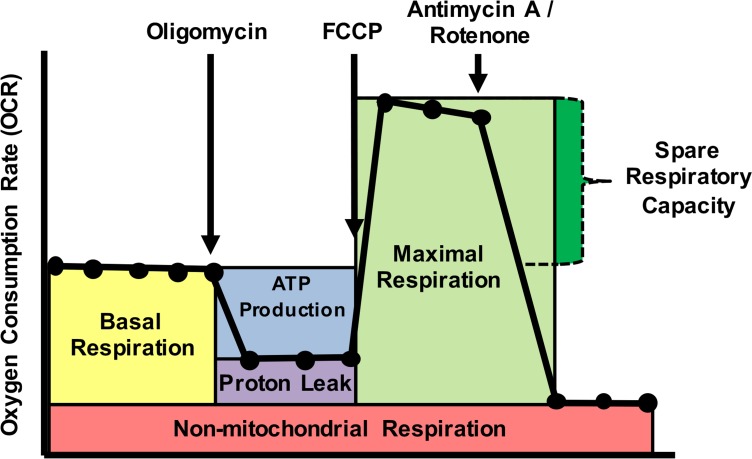
Schematic of oxygen consumption trace from extracellular flux analysis. Oligomycin, ATP synthase inhibitor; 1 μM fluoro-carbonyl cyanide phenylhydrazone (FCCP), ionophore which shuttles hydrogen ions; rotenone and antimycin A, complex I and III inhibitor respectively, are added to cells sequentially to assess mitochondrial respiration.

Cells were used immediately following isolation or after thawing rapidly in a 37°C water bath, then adhered at the numbers indicated onto wells of a poly-D-lysine coated XF96 plate. OCR and ECAR were measured using the mitochondrial stress test procedure in XF media (non-buffered DMEM containing 10 mM glucose, 4 mM L-glutamine, and 2 mM sodium pyruvate; note lack of buffer is absolutely required to measure pH drop that indicates ECAR) under basal conditions and in response to 3.5 μM oligomycin (Calbiochem), 1 μM fluoro-carbonyl cyanide phenylhydrazone (FCCP) (Enzo) and 14μM rotenone + 14 μM antimycin A (Enzo) with the XF96 Extracellular Flux Analyzer (Seahorse Bioscience) unless otherwise noted (Fig 1). Details of variations on standard XF protocols are outlined below.

### Analysis of metabolic profiles

To complete analysis of metabolic profiles generated by Seahorse, we developed a program that combined data from multiple plates and replicates to give metabolic profiles and OCR source calculations, with appropriate error and error propagation. The program is written in

Python with a simple graphical user interface. We provide this tool, which we have named SeaHORse Explorer (SHORE) free to the community under Gnu General Public License; it can be downloaded from GitHub at https://github.com/elizabethproctor/Seahorse-Analysis. The detailed description of program development below is summarized in Table 2.

**Table 2:** Summary of SeahHORse Explorer (SHORE) program development.

Application		Description
**Input**	File Origin	.xlsx derived from Wave assay results file (.asyr)
	Normalization	Cell number (100k) manually entered into an additional column in the “rate” tab of the.xlsx file
	File Format	.csv saved from the “rate” tab of modified.xlsx file
**Automated Data Cleaning / Quality Control**	Normalization	OCR value divided by the normalization value (e.g. cell number, total protein, number of mitochondria). Units are dependent on values used for normalization.
	Replicate Handling	Replicates are pooled for each experimental group and the median value for each OCR measurement is taken. The median is used because, (1) the distribution of values in theory is a bell curve, meaning the mean and median are equal, and (2) the median is resilient to outliers in the very probable case that there are outliers that would drastically affect the mean.
	Negative OCR values	Any assay wells with at least one negative OCR value are omitted from analysis for data integrity. Negative OCR values are not physically possible.
	Negative Spare Respiratory Capacity	Any wells with a negative spare respiratory capacity are omitted from analysis for data integrity. Physically, maximal respiration must be greater than or equal to basal respiration.
	Standardization across Plates	OCR values across multiple seahorse assays are baselined according to non-mitochondrial respiration, the least variable metabolic parameter across experimental groups.
**Parameter Calculations**	Statistics	The standard error of the mean is calculated for each value derived per experimental group.
	Basal Respiration	The mean OCR of the last 3 baseline data points minus the median of the 3 OCR data points after antimycin A and Rotenone addition.
	Basal OCR:ECAR	The mean OCR of the last 3 baseline data points divided by the mean of the ECAR of the last 3 baseline data points.
	ATP Production	The mean OCR of the last 3 baseline data points minus the mean of the 3 OCR data points after oligomycin addition.
	Non-Mitochondiral Respiration	The median of the 3 OCR data points after antimycin A and Rotenone addition.
	Proton Leak	The mean of the 3 OCR data points after oligomycin addition minus the median of the 3 OCR data points after antimycin A and Rotenone addition.
	Maximal Respiration	The highest of the 3 OCR values after the addition of FCCP.
	Maximal OCR:ECAR	The highest of the 3 OCR values after the addition of FCCP divided by the corresponding ECAR value.
	Spare Respiratory Capacity	The highest of the 3 OCR values after the addition of FCCP minus the mean OCR of the last 3 baseline data points.
**Output**	Wells Outputfile	.txt file containing OCR values and metabolic parameters for individual well/replicates categorized by experimental group
	Group Outputfile	.txt file containing OCR values and metabolic parameters + SEM for each experimental group

#### Program input

SHORE requires one input file, plus two optional parameters detailed below. The input file consists of the “Rate” tab of Seahorse output exported as an excel (.XLS) file then saved as a comma-separated values (.CSV) file. We added a column containing cell numbers to the CSV file to normalize OCR and ECAR in each well. For background wells, the input was “0”. Blank wells are assumed to be “0”. The program will not run without the input and cell number (or other designated by the investigator: protein input, etc.) normalization information. The first optional parameter the user may enter is the number of data measurements recorded during the mitochondrial stress test. We engineered the program to request the number of measurements; 14- (default) and 12-measurement options are currently implemented. The 14-measurement regime assumes a 5-3-3-3 outline, with five basal measurements taken, three following oligomycin, three following FCCP addition, and three following antimycin A/rotenone addition. The 12-measurement regime assumes a 3-3-3-3 outline, where the only difference from the 14-measurement regime is that three basal measurements are taken instead of five. The second optional parameter is an output filename, which will be used as the root for naming of output files (Program Output, below). If no output filename is indicated, “log” will be used as the root for naming output files.

#### Treatment of technical and biological replicates

Wells are grouped based on their entry by the investigator into the Seahorse software “Group” column to produce a single overall metabolic profile and set of metabolic parameter calculations. To account for plate-to-plate variation and batch effects, replicates within a group were baselined by their non-mitochondrial respiration (nMitoR), which was calculated as the median of the last three measurements in the profile following addition of antimycin A/rotenone (Calculation of Metabolic Parameters, below). We used the median value instead of the mean to discount skew from potential outliers without arbitrarily deleting data points. We chose nMitoR to baseline replicates due to its consistency across groups within a single plate. Note the operator can analyze the same data in two different ways: grouped by technical replicate or grouped by biological replicate, simply by renaming the wells in the group column.

#### Data cleaning

Replicates having OCR or ECAR measurement below zero were assumed to be in error and thus all data from such wells were automatically discarded and therefore excluded from calculations of metabolic profiles and parameters.

#### Calculation of measurement curves

After baselining by nMitoR (Treatment of Technical and Biological Replicates, above), we calculated metabolic profile measurement curves for each group as the median replicate value for each measurement. Because we expect a normal distribution of oxygen consumption rates at each measurement, in the case of no outliers or hardware errors resulting in artificially high or low measurements, the median value is equal to the mean value.

#### Calculation of OCR:ECAR ratio

The program calculates the ratio of OCR to ECAR at basal respiration and maximal respiration only when both values for a given measurement are above zero. If either value is zero or negative, all data from the well is excluded from calculations. The corresponding OCR:ECAR ratio from the median OCR well was retained as the ratio representing the corresponding group and measurement. After the calculation of measurement curves, the OCR:ECAR ratio at basal respiration for each group was reported as the mean of the ratios from measurements 3–5 (14-measurement regime) or measurements 2 and 3 (12-measurement regime), with appropriate error propagation (Handling and Propagation of Error, below). The OCR:ECAR ratio at maximal respiration for each group was reported as the ratio from the measurement with the highest OCR in the set of measurements 9–11 (14-measurement regime) or measurements 7–9 (12-measurement regime), with appropriate error propagation per below.

#### Calculation of metabolic parameters

The program uses the median value at each measurement to calculate metabolic parameters for each designated group: basal respiration (BR), ATP production (ATP), proton leak (PL), nMitoR, maximal respiration (MR), and spare respiratory capacity (SRC) (Fig 1). We also calculated these parameters on a well-by-well basis for breakdown by technical and/or biological replicates. These parameters are calculated as:

$$\begin{array}{l} nMitoR=median\left(M_{12},\;M_{13},\;M_{14}\right)\\ PL=median\left(M_{6},\;M_{7},\;M_{8}\right)\;\text{-}\;nMitoR\\ BR=mean\left(M_{3},\;M_{4},\;M_{5}\right)\;\text{-}\;nMitoR\\ ATP=BR\;\text{-}\;PL\\ MR=max\left(M_{9},\;M_{10},\;M_{11}\right)\;\text{-}\;nMitoR\\ SRC=MR\;\text{-}\;BR \end{array}$$

Where *M* is the set of 14 OCR measurements. For the 12-measurement regime, formulas were the same with *M* = *M*_*n-2*_, with the exception of:

$$BR=mean\left(M_{2},\;M_{3}\right)\;\text{-}\;nMitoR$$

In the group calculations, error is handled and propagated by standard rules, as discussed below.

#### Handling and propagation of error

Error in the calculation of measurement curves and metabolic parameters from technical and/or biological replicates was handled according to standard rules commonly defined as:

$$\begin{array}{l} Addition/subtraction:\;\varepsilon_{a+b}=\surd (\varepsilon_{a}{}^{2}+\varepsilon_{b}{}^{2})\\ Multiplication/division:\;\varepsilon_{ab}=\surd ({\left(\varepsilon_{a}/a\right)}^{2}+{\left(\varepsilon_{b}/b\right)}^{2}) \end{array}$$

We expect the distribution of replicate OCRs at each measurement to be Gaussian. For this reason, we calculated the error of each measurement in the group measurement curve as the standard error:

$$\varepsilon =\sigma /n^{2}$$

Where *σ* is the standard deviation of replicate OCRs, and *n* is the number of replicates.

For calculation of metabolic parameters (above), errors are propagated by standard rules per above except for calculations that use the median. Because these medians were taken from only three operands, the error is represented as the error of the operand chosen, without propagation.

#### Program output

The mitochondrial stress test analysis program outputs 2 files, named *wells_log*.*txt* (well-by-well calculations) and *groups_log*.*txt* (group calculations). If the optional file name parameter was used, “*log*” will be replaced by the text input by the user. Each file contained measurement curves and metabolic calculations, including OCR:ECAR ratios at basal and maximal respiration. In the group file, error was given for each calculation, as described above (Handling and Propagation of Error).

### IL-6 production

IL-6 production was measured by the DuoSet enzyme-linked immunosorbent assay (ELISA) kit according to the manufacturer’s instructions (R&D Systems).

## Results

### Titration of mitochondrial perturbation chemicals

**Fig 2:**
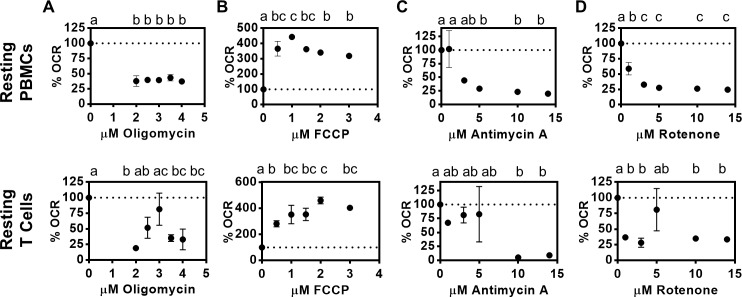
**Optimization of mitochondrial perturbation chemicals including (A) oligomycin, (B) FCCP, (C) antimycin A, and (D) rotenone for measurement of mitochondrial function in human immune cells.** Data is represented as the % of the basal OCR after addition of the indicated drug. N = 3. For all figures, values in the same panel with the same letter are statistically indistinguishable, values with different letters are statistically different as analyzed by 1-way ANOVA.

Mitochondria function varies amongst cell types. Therefore, one of the first steps for optimizing XF analysis is to titrate the automatically injected metabolic perturbation compounds that allow quantification of mitochondrial function. To optimize analysis of human PBMCs or CD4^+^ T cells (~700,000 cells per well), we first titrated the ATP synthase inhibitor oligomycin and the mitochondrial uncoupler FCCP from 0–4 μM, and the complex I/III inhibitors rotenone and antimycin A, respectively, from 0–14μM using the Seahorse XF Mito Stress Test program per manufacturer’s protocol. We analyzed the impact of the various perturbation chemicals on oxygen consumption at the initial reading following addition of the perturbation chemical. We found that OCR, normalized to cell number, was independent of oligomycin concentration for PBMCs, but minimal at 3.5–4μM for CD4^+^ T cells (Fig 2A). Maximum FCCP response was at 0.5–1.5μM for resting PBMCs and 1–3μM for resting CD4^+^ T cells (Fig 2B). Five-14 μM antimycin A/rotenone added individually equivalently inhibited mitochondrial function (Fig 2C and 2D). Given the titration outcomes, we concluded 3.5μM oligomycin, 1μM FCCP, and 14μM of antimycin A and rotenone were reasonable for subsequent analyses.

### Development of an algorithm to combine data generated by XF analysis

One significant limitation of XF analysis is the inability to objectively normalize and combine data over the multiple measurement points and multiple plates needed for the moderately sized studies that are required to establish cause/effect relationships between cytokine profiles [14] and human immune cell metabolism, especially in comparisons of samples from healthy and variously unhealthy subjects as required for T2D studies. A recent improvement in the Mito Stress Test Generator (an excel macro provided by Seahorse) added normalization over multiple plates to the previous version, but cannot analyze files with a custom run cycle, and still requires manual analysis as a quality control step. We therefore developed an algorithm in Python, SHORE, as described in the Methods section that automatically implements the following features over a virtually unlimited number of plates: normalization based on non-mitochondrial respiration to account for plate-to-plate variation, unlimited quantification of the OCR:ECAR ratio to detect shifts in fuel utilization, and use of replicate measurements to establish median as the value for each sample. Importantly, this approach captures information lost by normalized based on basal OCR [31]. Use of median appropriately eliminates impact of outlier points, samples from subjects with atypical responses due to unknown causes (subclinical infections, etc.), and points/outcomes that violate the laws of physics (for example, negative OCR). The features of SHORE and the conceptual advances for automated data cleaning and quality control are summarized in Table 2. SHORE significantly streamlined data analysis for further assay optimization described below.

### Titration of immune cell numbers

**Fig 3:**
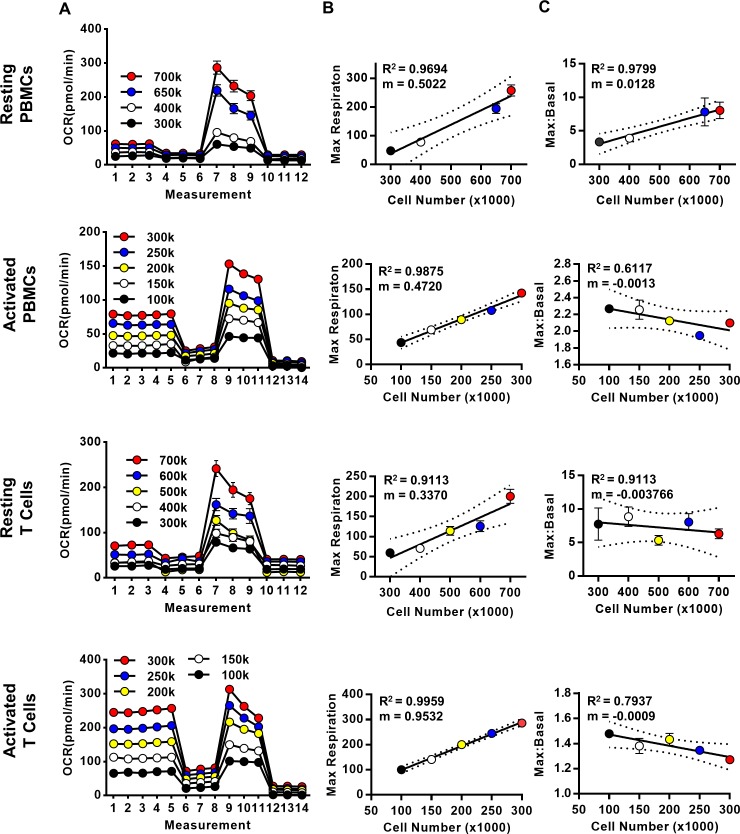
Titration of cell number per well for bioenergetics measurements in human PBMCs and CD4^+^ T cells. (A) Mitochondrial stress test seahorse profiles. (B). The relationship between the maximal respiration vs cell number is linear. (C) Ratio of the maximal respiration to the basal respiration is plotted against cell number. Resting PBMCs (N = 4), Activated PBMCs (N = 3), Resting T cells (N = 2), Activated T cells (N = 3).

One of the chronic limitations of biochemical analysis of human cells is that a limited number of cells can be harvested due to tissue volume concerns and/or safety considerations of the subjects, especially those with medical conditions or donating blood immediately prior to surgery. This limitation exacerbates the inherent variability in oxygen sensors, the need to perform XF in 3 or more technical replicates, and the multiplicity of samples generated by various stimulation conditions on cells from the number of individual subjects required for highly powered analyses. To identify the cell numbers needed to generate meaningful data, we plated up to 700,000 freshly isolated resting or stimulated PBMCs or purified CD4^+^ T cells from lean/healthy subjects in poly-D-lysine coated replicate wells of an XF96 plate, spun cells down to maximize adherence, and confirmed uniform distribution in the well. Note imaging attempts to quantitatively confirm cells were equivalently coating the wells were not useful, likely due to interference of plate materials. We immediately analyzed the immobilized cells using the XF program. Fig 3A shows measurable OCR in analysis of as few as 300,000 resting cells (top and third panels); preliminary studies with fewer resting cells yielded inconsistent profiles (not shown). Anti-CD3/CD28 stimulation increased OCR in both PBMCs and CD4^+^ T cells as expected, with reliable readings generated by as few as 100,000 cells per well (Fig 3A, second and bottom panels). Regression analyses showed a strikingly linear relationship between cell number and either maximal respiration (OCR; Fig 3B) or the maximal:basal OCR ratio (Fig 3C) for all conditions, indicating that analysis of as few as 300,000 resting or 100,000 stimulated cells validly measured mitochondrial respiration of PBMCs or human CD4^+^ T cells, and that the resolution of maximal from basal respiration is independent of cell number above these minimums.

### Technical considerations for cell preparation

**Fig 4:**
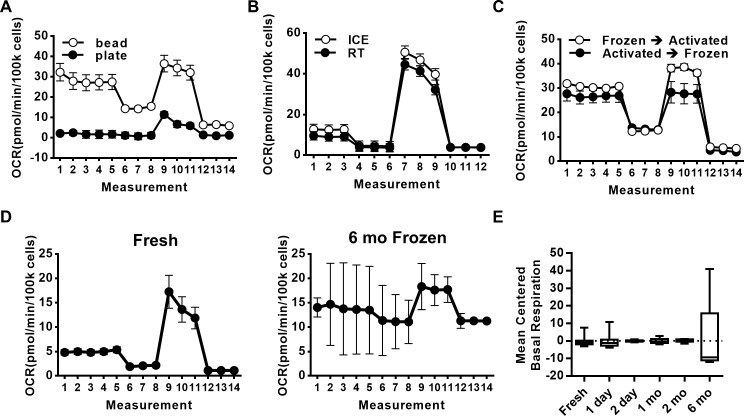
Technical considerations for performing flux analysis with primary human immune cells. (A) CD4 T cells were activated using anti-CD3/CD28 Dynabeads or a plate bound anti-CD3 and soluble anti-CD28 method for 40hrs. (N = 3) (B) Before running flux analysis, PBMCs were kept at room temperature (RT) or on ice during samples preparation (N = 3). (C) CD4+ T cells, isolated from PBMCs, were immediately activated for 40hrs and then frozen (Activated→Frozen) or CD4+ T cells, isolated from PBMCs, were first frozen, followed by T cell activation for 40hrs, then immediate flux analysis (Frozen→Activated) (N = 2). (D) Mitochondrial stress test profile from paired fresh (never frozen) PBMCs and PBMCs frozen for 6 months (N = 2). The mean centered basal respiration values of (E) resting PBMCs (N = 2) fresh or frozen for various amounts of time before flux analysis.

To optimize T cell stimulation conditions prior to XF analysis, we compared OCRs generated by traditional means of T cell stimulation (plate-bound αCD3 plus soluble αCD28) to more recently developed bead-bound T cell stimulation (see Methods), using cells from lean healthy subjects. Fig 4A shows that bead-bound T cell stimulation activated T cell oxygen consumption to a higher degree than the more traditional stimulation protocol. Bead stimulation was therefore used for all analyses herein. To further optimize the short-term cell storage that must occur while plates are being prepared, we compared results from stimulated cells that were washed then stored on ice (30–60 minutes) or instead rested at room temperature (~18°C) post-wash, prior to analysis of post-stimulation function. Fig 4B shows that stimulated PBMCs rested on ice or at room temperature similarly responded to mitochondrial perturbations based on OCR production. We conclude that bead-bound stimuli activate a more robust mitochondrial response, but short-term storage conditions following αCD3/CD28 stimulation are not critical for reproducible OCR analysis.

Given the challenges of recruiting human subjects to provide samples and the inevitable donor heterogeneity, and to minimize technical variation, many immunological studies freeze independent samples prior to parallel analyses. To test the impact of this logistically advantageous practice, we compared mitochondrial function in CD4^+^ T cells that were frozen, thawed, then subjected to XF analysis at 40 hrs of activation (Fig 4C, frozen → activated), to cells that were activated for 40 hrs, frozen, then thawed and immediately plated for XF analysis (Fig 4C, activated → frozen). OCR profiles of frozen → activated T cells more closely recapitulated published murine and human T cell profiles, and were also more similar to fresh PBMC OCR profiles than activated → frozen samples (compare Fig 4C to Figs 3A and 4D). To further test the impact of longer-term archiving on immune cell metabolic analysis, we froze resting cells from 1–180 days and compared OCR profiles to fresh cells. Fig 4D shows average profile from fresh PBMCs (left panel), which contrasts with the highly variable profile generated by PBMCs frozen for 6 months (right panel). Analyses of intermediate amounts of time frozen prior to activation/XF analysis are shown for PBMCs in Fig 4E. For technical reasons, paired samples for each time point were not available, thus findings are graphed as the mean of each group centered around zero, i.e. “mean centered basal respiration” measured prior to addition of oligomycin in this graph. We conclude that controlled freezing followed by rapid thaw after 6–8 wks at -170°C insignificantly impacts mitochondrial function, but that longer storage measurably affects precision of OCR determination. Notably, our freezing media (90% fetal calf serum/10% DMSO) maintained basal mitochondrial function somewhat longer than previously published conditions (50% RPMI, 40% autologous serum, 10% DMSO), despite use of controlled cooling in both studies [38].

### T cell metabolism increases over time

**Fig 5:**
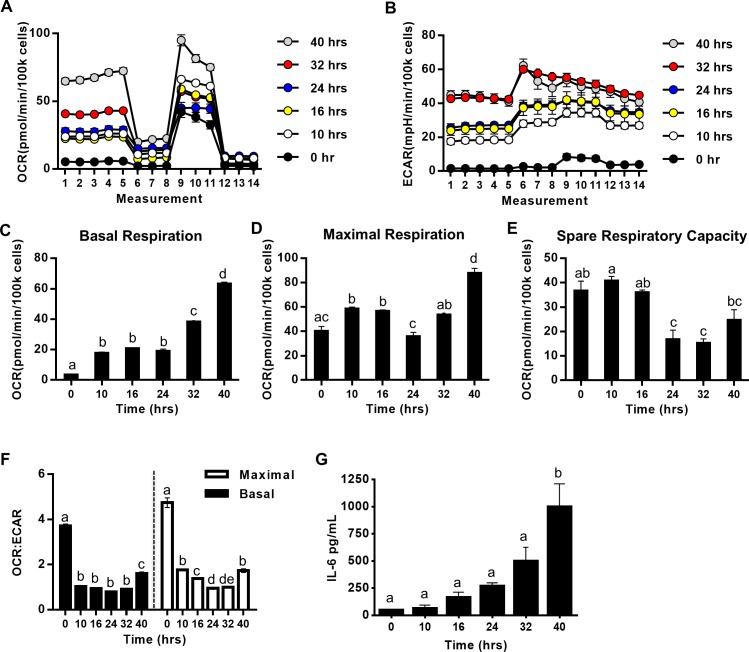
Bioenergetics Activation time course of CD4^+^ T cells. (A) OCR, (B) ECAR, (C) Basal Respiration, (D) Maximal Respiration, (E) Spare Respiratory Capacity, (F) OCR to ECAR ratio, and (G) secreted IL-6 of CD4^+^ T cells stimulated with anti-CD3/CD28 for indicated time. N = 3.

Our standard 40 hr stimulation protocol was based on our historical interest in IL-17A, which is first detectable 32 hrs after αCD3/CD28 stimulation, and robustly produced 40 hrs post-stimulation (data not shown and Ref. [10]). To focus specifically on T cells rather than PBMCs, and to determine whether T cell mitochondria continually increase aerobic and/or anaerobic respiration in the time leading up to significant cytokine production, we measured OCR and ECAR by T cells stimulated from 0–40 hrs. Fig 5A & 5B show both aerobic (OCR) and anaerobic (ECAR) respiration increase non-linearly over time, with basal OCR highest at 40 hrs, and ECAR maximal at 32 hrs. Closer inspection showed basal OCR transiently plateaued at 10–24 hrs (Fig 5C), the time during which T cells are preparing for the first post-stimulation division [35, 39]. CO_2_ is an unlikely contributor to the pH drop measured by ECAR, as indicated by lack of ECAR drop in response to Antimycin A/rotenone [31]. In contrast, maximal oxygen consumption increases in a potentially cyclical pattern (Fig 5D). SRC remains high up to 16 hrs post-stimulation then drops (Fig 5E), suggesting that pre-division T cells initially increase oxygen consumption while maintaining SRC until the first cell division. At later time points (i.e. after the first cell division) more oxygen is apparently being actively consumed rather than being held in reserve for maximal respiration. Analysis of the OCR:ECAR ratio at basal or maximal respiration (Fig 5F, black or white, respectively) showed unstimulated human CD4^+^ T cells were predominantly fueled by oxidative phosphorylation, but significantly decreased oxygen consumption and/or increased lactate production at all time points post-stimulation. This shift to aerobic glycolysis is consistent with single time-point results from previous rodent and human T cell studies [32, 40]. Basal OCR reached a nadir by 10 hrs post-stimulation, and maximal OCR was lowest at 24–32 hrs post-stimulation. Both basal and maximal OCR may begin to cycle back up at 40 hrs post-stimulation, although a longer time course would provide clarity on this possibility. Production of IL-6, a representative functional outcome of CD4^+^ T cell stimulation, increased logarithmically over time, despite the complex pattern of changes in OCR, ECAR or the OCR:ECAR ratio (Fig 5G). We conclude T cell respiration dynamically changes over time, yet fuels a consistent increase in IL-6 accumulation over the same time period.

### Analysis of mitochondrial function in diabetes

**Fig 6:**
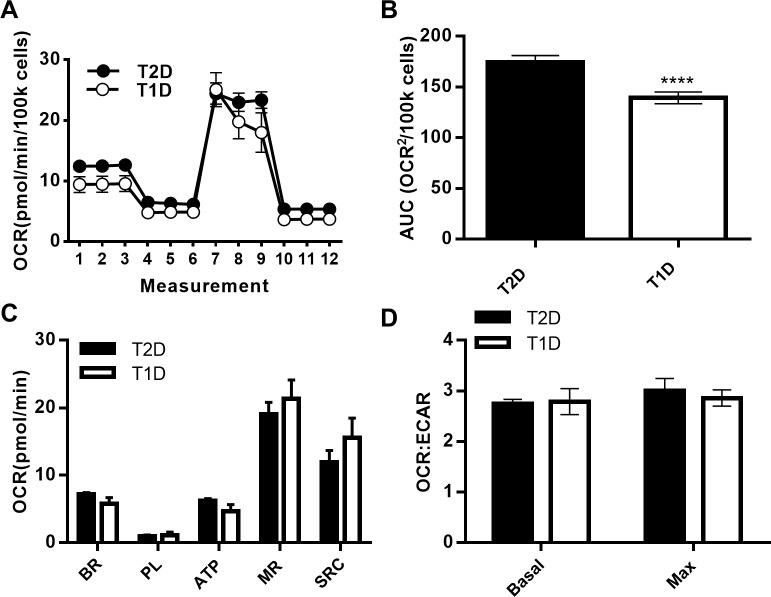
Mitochondrial respiration in PBMCs from T1D and T2D subjects. (A) OCR Mito Stress test Seahorse profiles and area under the curve analysis (B) for resting PBMCs from subjects with T2D (N = 11) or T1D (N = 6). (C) Metabolic parameters and (D) OCR:ECAR at basal and maximal respiration derived using the Seahorse analysis program. Unpaired two-tailed student’s *t* test for T2D vs T1D.

To pilot the utility of optimized conditions for XF analysis of human PBMCs, we first compared OCR profiles from resting cells provided by T1D and T2D subjects detailed in Table 1. We generated OCR curves for data from each biological replicate with SHORE (Fig 6A), then used these curves to calculate area under the curve in Prism. This approach showed that resting PBMCs from T2D subjects consumed more oxygen overall (Fig 6B) although ECAR was indistinguishable (data not shown). More detailed analysis of OCR in PBMCs responding to specific mitochondrial perturbations as quantified by SHORE showed that no single parameter differed despite low variation among biological replicate samples and N = 6–11 (Fig 6C). The OCR:ECAR ratio at either basal or maximal respiration was similar in PBMCs from both groups of subjects (Fig 6D). We conclude basal and maximal oxidative phosphorylation is overall similar in resting immune cells from T1D and T2D subjects.

### Comparison of mitochondrial function by resting human T cells and B cells

**Fig 7:**
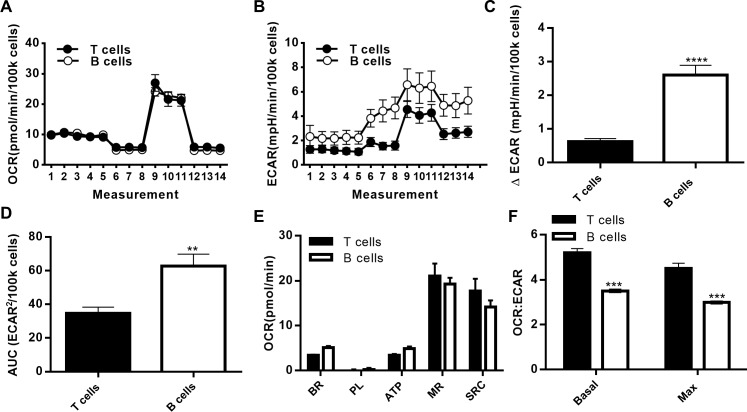
Mitochondrial respiration in T and B cells from healthy lean donors. (A) OCR and (B) ECAR Mito Stress test Seahorse profiles for resting T and B cells from the same healthy individuals (N = 3). (C) Change in ECAR after addition of oligomycin. Data is the average of measurements 6–8 from panel B minus measurements 3–5. (D) Area under the curve analysis of ECAR profile. (E) Metabolic parameters and (F) OCR:ECAR at basal and maximal respiration using SHORE. Differences in panels C-F are determined by paired two-tailed student’s *t* test.

Given that B cells are extremely limited in human blood (about 10% as plentiful as T cells), and concentrated sources of B cells such as leukopacks have quality inconsistencies (data not shown), we tested the possibility that conditions optimized for T cells might suffice for preliminary analysis of circulating B cells. OCR profiles for resting T cells and B cells analyzed under the same conditions (numbers, perturbation chemicals etc.) showed both types of lymphocytes from the same set of lean individuals consume oxygen similarly (Fig 7A). In contrast, overall ECAR and ΔECAR, the latter in response to oligomycin, was higher in B cells compared to T cells, with the more robust response to oligomycin indicating that B cells more readily switch to anaerobic glycolysis following blockade of ATP synthase compared to T cells (Fig 7B–7D). Although OCR after each perturbation was indistinguishable between T cells and B cells (Fig 7E), the OCR:ECAR ratio was lower in B cells compared to T cells under both basal and maximal respiration conditions (Fig 7F), consistent with previously published results from a comparison of murine T and B cells [16, 41]. Overall, the data demonstrate the practicality of using conditions optimized for a plentiful cell type as a starting point for unbiased analyses of a closely related cell type that is available in limited numbers.

## Discussion

We have developed a reproducible method for XF96 analysis of human PBMCs and CD4^+^ T cells. Perhaps most importantly, we have published a free downloadable XF analysis tool, SHORE, which has automated quality control and can handle data from an unrestricted number of samples exclusive of operator bias. SHORE is a critical advance for analysis of outcomes from the large number of samples required to make strong conclusions given the known variability of human samples, and the importance of analyzing outcomes from multiple treatments in parallel. We also generated preliminary evidence that our systematic approaches will facilitate analysis of both PBMCs, which are relatively plentiful, and lymphocytes, which can be available in relatively low numbers. The ability to ascertain detailed outcomes from a relatively limited number of cells is an important advance given that the Seahorse platform has only been somewhat miniaturized since the original design for cell line usage. Our work builds on earlier detailed descriptions of Seahorse protocols and the commercially available Wave and Mito Stress Test Generator programs [31, 42] that although initially useful, needed to be modified to more fully exploit the technology.

The cell number requirements and mitochondrial perturbation concentrations determined herein are consistent with published work that showed XF analysis requires 75,000–600,000/well of freshly isolated human peripheral blood cells to measure oxygen consumption profiles [42–44], with similar numbers analyzed in experiments on activated immune cells [32, 35, 42, 43]. Given the sensitivity of oxygen sensors, it is often necessary to run ≥3 replicate wells to generate meaningful data. Taken together, the number of cells and technical replicates needed for XF significantly increases the analytical burden of combining data from technical replicate wells over the multiple plates required for biological replicates, or due to the practicalities of run timing. Our cell number titration and linearity of response over multiple numbers of cells confirms the need for relatively large numbers of cells/well, and the need for fewer cells following metabolic activation with standard stimuli. Analysis of impacts of T cell activation methods and cellular storage conditions over the short-term (on ice versus room temperature) or long-term (-170°C) solve some of the previously identified practical problems with human PBMC analysis [44]. The studies on time course of metabolic action showed unexpected non-linear patterns of oxygen consumption after stimulation of our mixed naïve/memory CD4^+^ T cell population. The potentially cyclical nature of maximal respiration and SRC strikingly differed from the logarithmic increase in T cell function, at least as measured by IL-6 accumulation. Taken together, these data indicate that more detailed longitudinal combinations of XF and cytokine production (rather than cytokine accumulation as measured herein), combined by SHORE and the multivariate cytokine analyses that we previously published [14], may reveal novel insights into the dynamic nature of lymphocyte regulation by respiratory pathways.

The metabolic profile comparisons between T1D and T2D cells, or T cells and B cells presented herein used resting cells, but nevertheless illustrated the power of XF analysis by our newly developed SHORE analytical tool. Given the long-held appreciation that stimulated immune cells increase fuel utilization as recapitulated in Fig 2, it is highly likely that differences will be more obvious upon immune cell activation in future studies. We also noted that PBMC metabolic profiles are similar to CD4^+^ T cell profiles under resting conditions. This result is perhaps expected because T cells comprise about 60% of PBMCs. However, metabolic profiles diverge between αCD3/CD28-stimluated PBMCs and CD4^+^ T cells. The most obvious example of this outcome is for activated PBMCs and T cells in Fig 2, which shows that basal respiration is higher and SRC is significantly lower for purified T cells compared PBMCs. The cell types and/or cell-cell interactions responsible for the differences in oxygen consumption of PBMCs compared to purified T cells was not addressed by our analyses. Regardless, our findings raise the possibility that functionally important interactions between T cells and other PBMC cell types, evidenced by work in T2D [9, 10, 14], are critical determinants of T cell metabolism that may mechanistically explain these functional readouts.

Taken together, the 1. ability to reproducibly quantitate different sources of oxygen consumption, with slight variations on program predicted to be equally useful for other XF mitochondrial perturbation kits; and 2. analysis of virtually infinite sample numbers and technical replicates of primary human cells, will significantly increase the utility of XF analysis as a tool to identify drivers of specific immune cell functions. Given the ongoing development of metabolic regulatory drugs, and perhaps most importantly food and food derivatives, the identification of these processes holds high promise towards low-risk methods to normalize immune cell function in a variety of common diseases including obesity and T2D.
